# Long-term Qipian® administration confers resistance to airway inflammation in OVA-induced asthmatic mice

**DOI:** 10.3389/falgy.2026.1746439

**Published:** 2026-02-26

**Authors:** Huiying Wang, Ru-Yi Chen, Jin-Jin Shi, Guan-Jun Yang, Jiong Chen

**Affiliations:** 1 Department of Allergy and Clinical Immunology, The Second Affiliated Hospital of Zhejiang University School of Medicine, Hangzhou, Zhejiang, China; 2 School of Marine Sciences, Ningbo University, Ningbo, China

**Keywords:** CD103^+^ DCs, CD4^+^CD25^+^Foxp3^+^ Treg cells, gut microbiota, long-term Qipian® administration, OVA-induced asthma

## Abstract

**Background:**

Qipian® is an immunomodulatory agent with established short-term benefits in allergic asthma, but its long-term effects remain unclear. This study aimed to investigate its potential to attenuate the development of asthma in a murine model and to elucidate its underlying mechanisms.

**Methods:**

Mice received oral Qipian® for 3 months prior to establishment of an ovalbumin (OVA)-induced asthma model. Samples (lung tissues, blood, bronchoalveolar lavage fluid, and feces) were collected. Analyses included quantification of eosinophils, immunoglobulins, and Th1/Th2 cytokines. Lung mucus was assessed via periodic acid-Schiff staining; dendritic cell and regulatory T (Treg) cell populations were characterized by flow cytometry; and gut microbiota was profiled via 16S rDNA sequencing. Asthmatic symptoms were scored concurrently.

**Results:**

Long-term Qipian® administration (LTQA) effectively reduced OVA-induced asthmatic symptoms, airway inflammation, inflammatory cell infiltration, and mucus hypersecretion. LTQA restored the Th1/Th2 balance by reducing IL-4, IL-5, and IL-13, while elevating the expression of IFN*γ* and IL-10. Furthermore, LTQA was associated with the expansion of Tregs and CD103^+^ dendritic cells, reduction of OVA-elevated neurokinins [neurokinin A (NKA), neurokinin B (NKB)], and increased abundance of *Lactobacillus*.

**Conclusion:**

This study indicates that LTQA may confer resistance to allergic airway inflammation by modulating immune responses and gut microbiota supporting the lung–gut axis as a promising target for novel clinical approaches in asthma management.

## 1 Introduction

Asthma is a pervasive chronic airway inflammation affecting approximately 1%–9% of the global population (1). It is characterized by recurrent episodes of respiratory symptoms such as cough, dyspnea, and wheezing, along with reversible airflow restriction, which finally leads to the remodeling and narrowing of airways (2). The typical symptom of asthma is the airway inflammation involving multiple inflammatory cells such as mast cells, eosinophils, dendritic cells, and the Th2 cells-dominated immune response. Conventional anti-inflammatory therapies, such as inhaled glucocorticosteroids and the newly emerging biologics, have improved the control of asthma dramatically. However, they are helpless in curbing the rapid rise of morbidity. Preventing the occurrence and progression of asthma continues to remain a substantial challenge.

Increasing evidence suggests that immune modulatory strategies aimed at changing adaptive immune imbalance might be the best way to prevent asthma. Asthma is a multifaceted disease characterized by intricate interactions between genetic predisposition and environmental influences. For example, early exposure to environmental microbes could reduce the incidence of asthma, as demonstrated in the 2016 study of Amish and Hutterite children (3). Similarly, the uptake of bacterial lysates (BLs) has shown promise in the therapy of asthma, as they can modulate the immune response and potentially reduce airway inflammation (4–9). BLs are derived from inactivated or disrupted bacterial cells, which contain various components such as proteins, nucleic acids, and cell wall fragments. These components have the ability to stimulate the immune system and promote a balanced immune response (10, 11). These interventions have demonstrated effectiveness in reducing the frequency and severity of asthma, improving lung function, and decreasing the reliance on rescue medication (12). Our previous study demonstrated that Qipian® inhibits established inflammation of ovalbumin (OVA)-induced asthma in a mouse model (7). The exact mechanisms by which BLs exert their therapeutic effects in asthma are not fully understood. However, it is believed that they can modulate the immune response through several mechanisms. BLs demonstrate a multifaceted immunomodulatory capacity, characterized by the promotion of anti-inflammatory cytokines, notably interleukin-10 (IL-10), alongside the inhibition of pro-inflammatory cytokines like IL-4 and IL-5. They also bolster the function of regulatory T cells (13). Beyond their immunomodulatory effects, BLs exert influence over the composition and functionality of the gut microbiota. There is growing evidence suggesting a link between gut dysbiosis and the development of asthma (8). BLs may restore a healthy gut microbiota balance, which in turn can positively impact immune regulation and reduce allergic inflammation (14). Yet, whether these underlying mechanisms also prevent the development of asthma is still unknown.

Qipian® is a lyophilized, fractionated alkaline extract derived from three distinct genera, namely, *Neisseria catarrhalis*, *Bacillus subtilis*, and *Staphylococcus albicans* (7). With over 30 years of clinical use in China, it has been employed as a therapeutic intervention for chronic bronchiolitis. Notably, Qipian® has exhibited efficacy in ameliorating respiratory symptoms such as cough and sputum production by reducing the duration of respiratory infections while concurrently augmenting the production of immunoglobulins (Igs), thereby exerting immunomodulatory effects (7, 15, 16). A retrospective cohort study has provided evidence suggesting that Qipian® has a beneficial effect in alleviating pediatric asthma (17). A recent study also showed that short-term Qipian® treatment could alleviate OVA-induced asthma in mice (7). However, the preventive effect and potential role of long-term Qipian® administration (LTQA) in asthma are unknown. Here, an OVA-induced asthma model was used to assess the preventive effect of LTQA on asthma and to uncover its potential mechanism in order to explore a novel preventive strategy for asthma control.

## 2 Materials and methods

### 2.1 Animals

A total of 60 female Balb/c mice, aged 6 weeks, were obtained and housed in a sterile rodent facility at the Laboratory Animal Center of Ningbo University. All procedures for experiments with animals followed the rules and regulations set by the Animal Care and Use Committee at Ningbo University.

Ethical approval for the study was granted under Approval No.11781.

### 2.2 Establishment of an OVA-induced asthmatic mouse model and sample collection

**Figure 1:**
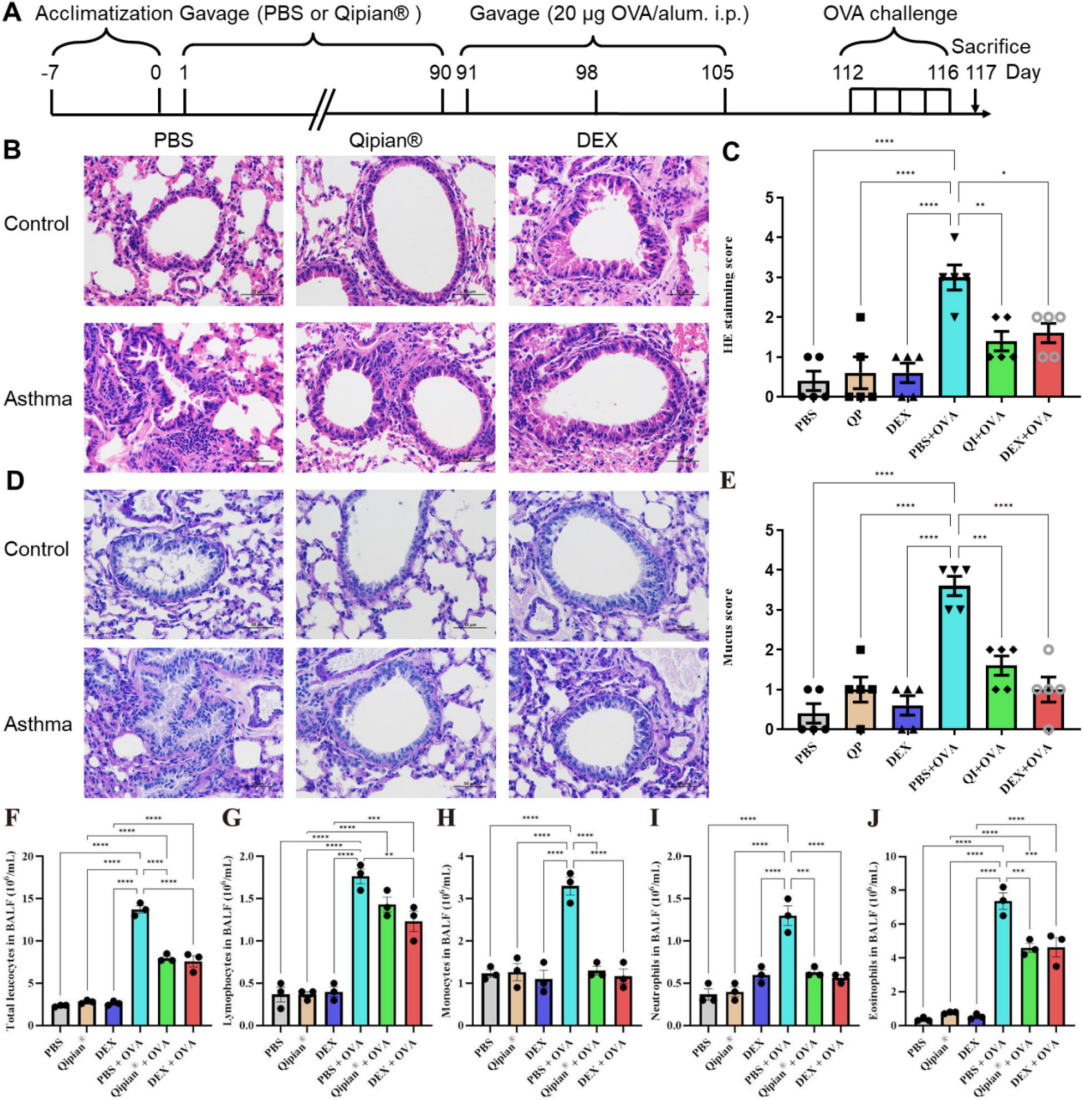
LTQA alleviates asthma symptoms in OVA-induced asthmatic mice. **(A)** Schematic of the experimental OVA-induced asthma model and drug administration timeline. **(B)** Representative H&E-stained lung sections from each treatment group. **(C)** Quantitative airway inflammation scores (mean of five visual fields per sample). **(D)** Representative periodic acid–Schiff (PAS)-stained lung sections showing mucus production. **(E)** Quantitative mucus index scores (mean of five visual fields per sample). **(F–J)** Differential leukocyte counts in bronchoalveolar lavage fluid (BALF): **(F)** total leukocytes, **(G)** lymphocytes, **(H)** monocytes, **(I)** neutrophils, and **(J)** eosinophils. Statistical significance is denoted as *p < 0.05, **p < 0.01, ***p < 0.001, and ****p < 0.0001.

The experimental mice were split into six groups, each containing 10 mice. Two groups received a daily oral dose of 25 mg/kg Qipian® for 90 days. The protocol outlined in Figure 1A was followed to establish the asthmatic model. On day 91, three groups of mice—including with one group administrated Qipian®—were underwent sensitization through intraperitoneal injection of a gel solution with ratio of mass of OVA and Al(OH)3 of 20 μg: 2 mg in 0.9% NaCl solution, as previously described, with minor modifications (Figure 1A) (18). Subsequently, 3% OVA in normal saline solution was administered via nasal drip once daily from day 112 to day 116 using a 200 μL pipette. The other three groups were given injections of PBS, administered intraperitoneally and intranasally, as control groups. The dose selection for both Qipian® and the dexamethasone (DEX) was in accordance with our previous work (7). Among the groups, the first group was the PBS control mice. The second and fifth groups received a daily oral dose of 25 mg/kg Qipian® (lasting for 3 months) with the OVA-induced asthma model and PBS control, respectively. The fourth group was the OVA-induced asthma group treated with PBS. The third and sixth groups were injected with 1.0 mg/kg DEX daily from day 112 to day 116 before OVA challenge and PBS control, respectively (Figure 1A). After a 24-h interval, all mice were euthanized with a high concentration of isoflurane (5% in oxygen or medical air) for a minimum of 2 min after cessation of breathing. Samples of blood, lung tissues, and bronchoalveolar lavage fluid (BALF) were collected for further analysis. Meanwhile, mouse feces samples from every group were collected and stored for further 16rDNA sequencing. All fecal samples were snap-frozen and stored at −80°C until DNA extraction. The storage period did not exceed 1 week, and samples underwent no freeze–thaw cycles to ensure microbiome integrity.

### 2.3 Asthmatic score criteria

The study recorded the asthmatic behavior scores of each mouse based on the number of nasal scratching, sneezing frequency, and degree of wheezing after nasal dripping. The criteria followed previous reports (Table 1) (7, 19).

**Table 1:** Criteria and scoring system for assessing asthma-like behaviors in mice.

Behaviors	Records	Scores
Sneezing	0	0
	4	1
	4–10	2
	>11	3
Nasal scratching	No nasal scratching	0
	More than one nasal scratching	M
	Mild nasal scratching	2
	Frequent nasal scratching	3
Wheezing	No wheezing	0
	Shortness wheezing	1
	Significant wheezing	2
	Dearth by wheezing	3

### 2.4 BALF collection and cell counting

After the mice were euthanized, bronchoalveolar lavage was performed by instilling 1 mL of PBS into the lungs three times, following established protocols (20). The resulting BALF was collected and subsequently centrifuged. The pellet obtained from centrifugation was resuspended in ice-cold PBS for further analysis. The supernatant was utilized to assess changes in cytokines (IL-4, IL-5, IL-13, IL-10, IL-33, and IFNγ) and immunoglobulins (Igs, IgG, IgA, IgE, and OVA-IgE), while the pellet was employed to determine the proportion of different immune cells. The cell count in each sample was measured using the hematology analyzer XS 500i (Sysmex, Kobe, Japan).

### 2.5 Collection of serum and lung homogenates

Serum preparation: Blood samples were collected and incubated at 4°C overnight to allow complete clotting. Serum was separated by centrifugation at 12,000 × g for 10 min at 4°C, aliquoted, and stored at −80°C until analysis of immunoglobulin levels (total IgG, IgA, IgE, and OVA-specific IgE). Lung tissue homogenization: For tachykinin extraction, lung tissues were weighed and homogenized on ice in RIPA lysis buffer (1:10 w/v) supplemented with 1× Halt™ Protease Inhibitor Cocktail (Thermo Fisher, #78430) using a TissueLyser II (Qiagen) for 2 min at 25 Hz (7). The homogenates were centrifuged at 10,000× g for 15 min at 4°C. The resulting supernatant was collected, and its total protein concentration was quantified using the Pierce™ BCA Protein Assay Kit (Thermo Fisher, #23225) according to the manufacturer's instructions. Tachykinin [SP, neurokinin A (NKA), neurokinin B (NKB)] levels were then measured in the normalized supernatants (21, 22).

### 2.6 Enzyme-linked immunosorbent assay

Specific antibodies were applied to 96-well plates and left to incubate overnight at 4°C. Following that, the specimens were placed onto the dishes and left to incubate for a duration of 2 h at a temperature of 37°C. After that, secondary antibodies conjugated with horseradish peroxidase were introduced and left to incubate for an hour. To observe the enzyme's reaction, a solution of tetramethylbenzidine substrate was added and the change in color was identified using a SpectraMax Paradigm multimode reader from Molecular Devices, CA, USA. The plates were washed three times following each step. Prior to quantification, absorbance at 450/595 nm was determined after acidification with 0.18 M H_2_SO_4_. Details of ELISA kits are provided in Table 2.

**Table 2:** The ELISA kits used in this study.

Kit name	Manufacturer	Catalog number
Mouse IL-4 ELISA Kit	Mult Sciences	EK204
Mouse IL-5 ELISA Kit	Mult Sciences	EK205
Mouse IL-10 ELISA Kit	Mult Sciences	EK210
Mouse IL-13 ELISA Kit	Mult Sciences	EK213
Mouse IL-33 ELISA Kit	Mult Sciences	EK233
Mouse IgG ELISA Kit	Mult Sciences	EK271
Mouse IgA ELISA Kit	Mult Sciences	EK274
Mouse IFN*γ* ELISA Kit	Mult Sciences	EK280
Mouse IgE ELISA Kit	Kote	KT2056-A
Mouse OVA-specific IgE ELISA Kit	Kote	KT2291-A
Mouse NKA ELISA Kit	Kote	KT30651-A
Mouse NKB ELISA Kit	Kote	KT30655-A
Mouse SP ELISA Kit	Kote	KT2445-A

### 2.7 Lung histopathological assay

The lateral lobe of the left lung were embedded in a sagittal plane orientation in paraffin, sectioned at a thickness of 4–5 µm, and stained with hematoxylin–eosin or periodic acid–Schiff (PAS) according to standard protocols (23). Slides were examined under a light microscope to determine the inflammatory cell infiltrates, as well as mucus hypersecretion. Lung inflammation severity was measured on a scale of 0–3, as described in a previous study (24). Mucus production was assessed visually in 10 randomly chosen microscopic fields, with a scoring system ranging from 0 to 4 based on PAS staining intensity. A score of 0 meant no visible PAS stain, 0.5 indicated a low level of staining, 1 represented about a quarter of the bronchial space filled with mucus, 2 indicated half, 3 indicated three-quarters, and 4 indicated full mucus coverage (7). Each group was scored on a minimum of three non-serial sections per mouse to ensure the stability of the experimental results. All semiquantitative histological analyses (inflammation and mucus scores) were conducted by a researcher blinded to sample identity and treatment group. For each mouse, a minimum of two non-serial lung sections were scored, and the average score per animal (biological replicate, *n* = 5 mice/group) was used for statistical analysis.

### 2.8 Single-cell preparation

The methodology described here outlines a rigorous process for preparing lung tissues for downstream analysis. Lung tissues were fragmented followed by digestion using Dulbecco's Modified Eagle Medium (DMEM) digestion media to release individual cells. The resulting suspension was passed through a nylon mesh strainer to eliminate debris and aggregates, yielding single-cell suspensions. RBC lysis buffer further ensures purity by removing residual red blood cells. Cells were then washed with DMEM medium supplemented with fetal bovine serum to help maintain cell viability. Detection of alterations in Treg cells and CD103^+^ dendritic cells (DCs) after treatment was made possible by staining using specific biomarkers.

### 2.9 Flow cytometric analysis

Single-cell suspensions were prepared at a density of 2 × 10^6^ cells/mL. To mitigate non-specific antibody binding, cells were blocked with anti-CD16/32 Fc receptor antibody (eBioscience) for 15 min at 4°C. Surface staining was performed using fluorochrome-conjugated antibodies against I-A/I-E (M5/114.15.2), CD11b (M1/70), CD11c (N418), and CD103 (2E7), as previously outlined (25). For regulatory T cell (Treg) analysis, cells were stained with anti-CD4-FITC and anti-CD25-APC (BD Biosciences), followed by fixation, permeabilization (eBioscience kit), and intracellular staining with anti-Foxp3-PE. Fluorescence-minus-one and isotype controls were included in each experiment to accurately set gates and determine non-specific background. Compensation was applied using single-stain controls. Cells were analyzed on a Beckman Coulter CytoFLEX™ (CytoFLEX). Standardized gating strategies were employed: Cells were gated on live/singlets → lymphocytes (for Tregs) or live/singlets → CD11c + (for DCs) → subsequent markers. For each biological replicate (*n* = 3 mice/group), data from a minimum of 50,000 live, single-cell events were collected and analyzed using FlowJo v10.8.1 (7).

### 2.10 Gut microbiota test

Fecal samples were collected, immediately flash-frozen in liquid nitrogen, and stored at −80°C until processing. Microbial genomic DNA was extracted using the HiPure Stool DNA Kit (Magen) according to the manufacturer's instructions. Negative controls (sterile water) were included during extraction and library preparation to monitor contamination. The V3–V4 hypervariable regions of the bacterial 16S rRNA gene were amplified with barcoded primers (341F/806R) and sequenced on an Illumina NovaSeq 6000 platform (LC-Bio, China) using 2 × 250 bp paired-end chemistry.

Bioinformatic processing: Raw paired-end reads were quality-filtered using Fastp (v0.23.2) to remove low-quality bases (Q-score <20) and adapters. Amplicon sequence variants (ASVs) were inferred using the DADA2 pipeline in QIIME2 (v2022.8), which includes error correction, chimera removal, and taxonomic assignment against the SILVA 138 database. The final ASV table was rarefied to a depth of 10,000 sequences per sample for all downstream analyses. Alpha diversity (Shannon, Simpson indices) and beta diversity (Bray–Curtis dissimilarity) were calculated (26). Principal coordinates analysis (PCoA) was performed and visualized using the *ggplot2* package in R (27). Differential abundance was assessed using linear discriminant analysis effect size (LEfSe) with a logarithmic LDA score threshold of >2.0 to identify biomarker taxa and *p*-values were adjusted for multiple testing using the Benjamini–Hochberg false discovery rate (FDR) method (28, 29). Venn diagrams were generated to visualize shared ASVs. Predictive functional profiling of the microbiota was performed using Tax4Fun2, mapping 16S rRNA gene sequences to KEGG orthologs. Notably, these functional predictions are inferred from taxonomic data and represent putative metabolic potential.

Statistical analysis: Alpha and beta diversity metrics were calculated in QIIME2. Group differences in beta diversity were tested with PERMANOVA (999 permutations) using the *vegan* package in R. Differential ASV abundance was determined using the ANCOM-BC package with FDR correction. LEFSe was used for biomarker discovery (LDA score >2.0, FDR-adjusted *p* < 0.05). Functional potential was predicted from 16S data using Tax4Fun2 (30).

### 2.11 Statistical analyses

All statistical analyses were performed using GraphPad Prism 7.0 software (La Jolla, CA, USA). The information is presented as averages with the standard error of the mean. Student's *t*-test was used for data that followed a normal distribution, whereas the Mann–Whitney *U* test was employed for data that did not follow a normal distribution. Lung inflammation scores and BALF cell counts were analyzed using two-way analysis of variance with Sidak's multiple comparisons test. Statistical significance was determined as a *P* value of less than 0.05. Selection of the appropriate statistical techniques depended on the characteristics of the data to ensure precise group comparisons and the validity of experimental results.

## 3 Manuscript formatting

### 3.1 LTQA relieves symptoms of lung features in OVA-induced asthmatic mouse model

OVA sensitization and challenge significantly induced airway inflammation, characterized by eosinophil infiltration and mucus accumulation in lung tissues. LTQA significantly alleviated these pathological features, as indicated by reduced inflammatory cell infiltration in H&E-stained sections and decreased mucus production (Figures 1B–D). Histological examination demonstrated that LTQA suppressed airway inﬂammation to a degree comparable to DEX. Furthermore, cytological analysis of BALF revealed that LTQA markedly reduced the OVA-induced increase in eosinophil count (Figures 1F,J).

### 3.2 The effects of LTQA on humoral immune response

**Figure 2:**
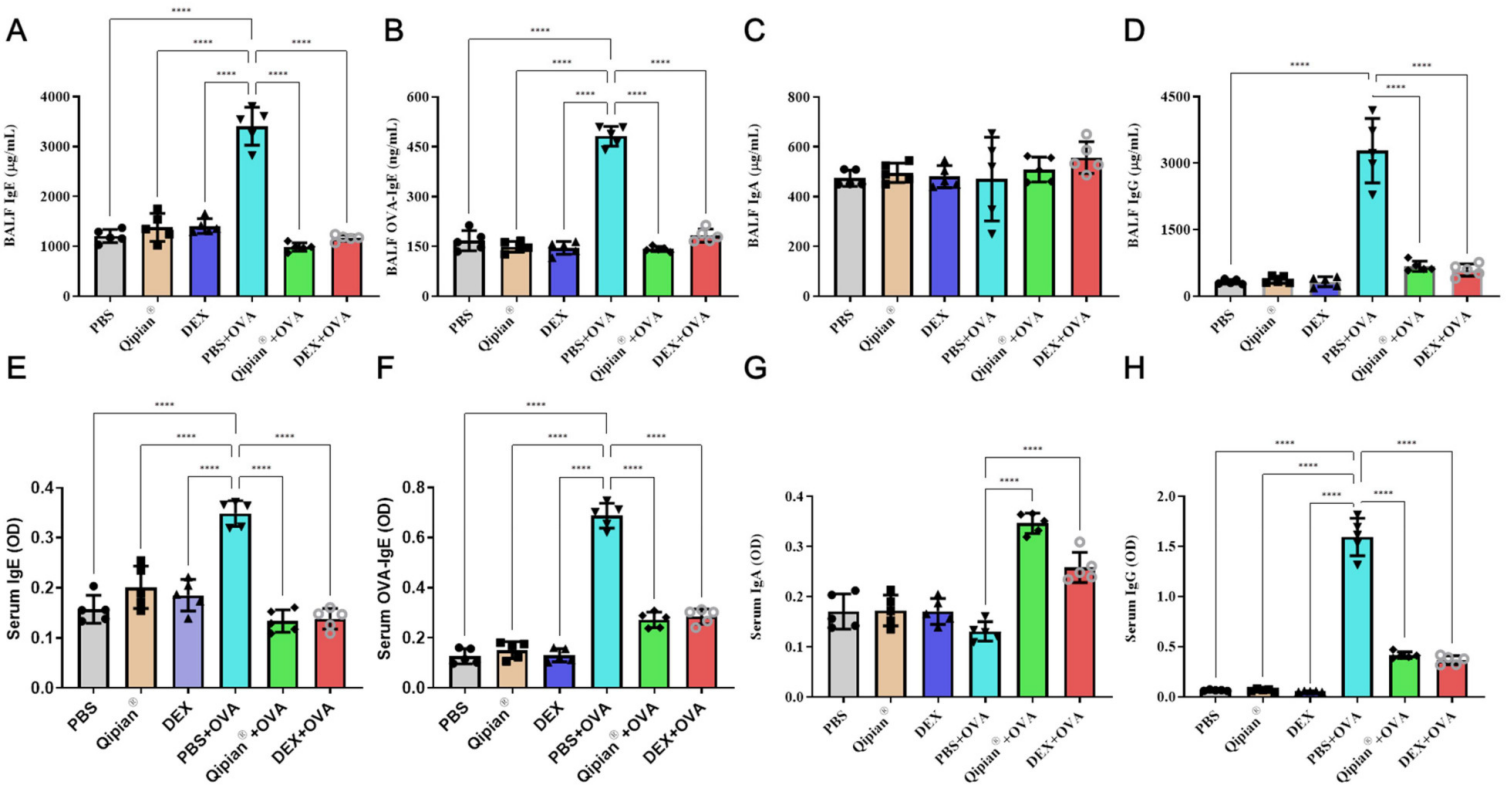
LTQA modulates immunoglobulin levels in the airways and serum. Immunoglobulin concentrations were measured in BALF and serum following OVA challenge and LTQA treatment. Shown are levels of **(A,E)** total IgE, **(B,F)** OVA-specific IgE, **(C,G)** IgA, and **(D,H)** IgG. Statistical significance is denoted as *p < 0.05, **p < 0.01, ***p < 0.001, and ****p < 0.0001.

The humoral immune response plays a crucial role in the onset and progression of asthma, involving activation of B cells and DCs to produce immunoglobulins (Igs) such as IgE and IgG upon exposure to exogenous antigens. In our model, OVA sensitization and challenge resulted in a significant elevation of total IgE (Figures 2A,E), OVA-specific IgE (Figures 2B,F), and IgG (Figures 2D,H) in both serum and BALF. LTQA significantly reduced the levels of these Igs, indicating its suppressive effect on OVA-induced humoral response (Figures 2A,B,D–F,H). In contrast to previous findings on short-term treatment with Qipian® (7), LTQA showed minimal inhibitory effect on IgA secretion in serum and BALF (Figures 2C). Notably, it increased IgA levels in BALF following OVA challenge (Figure 2G). These findings suggest that LTQA differentially modulates systemic and mucosal humoral immunity.

### 3.3 Effects of LTQA on cytokine profiles in BALF

**Figure 3:**
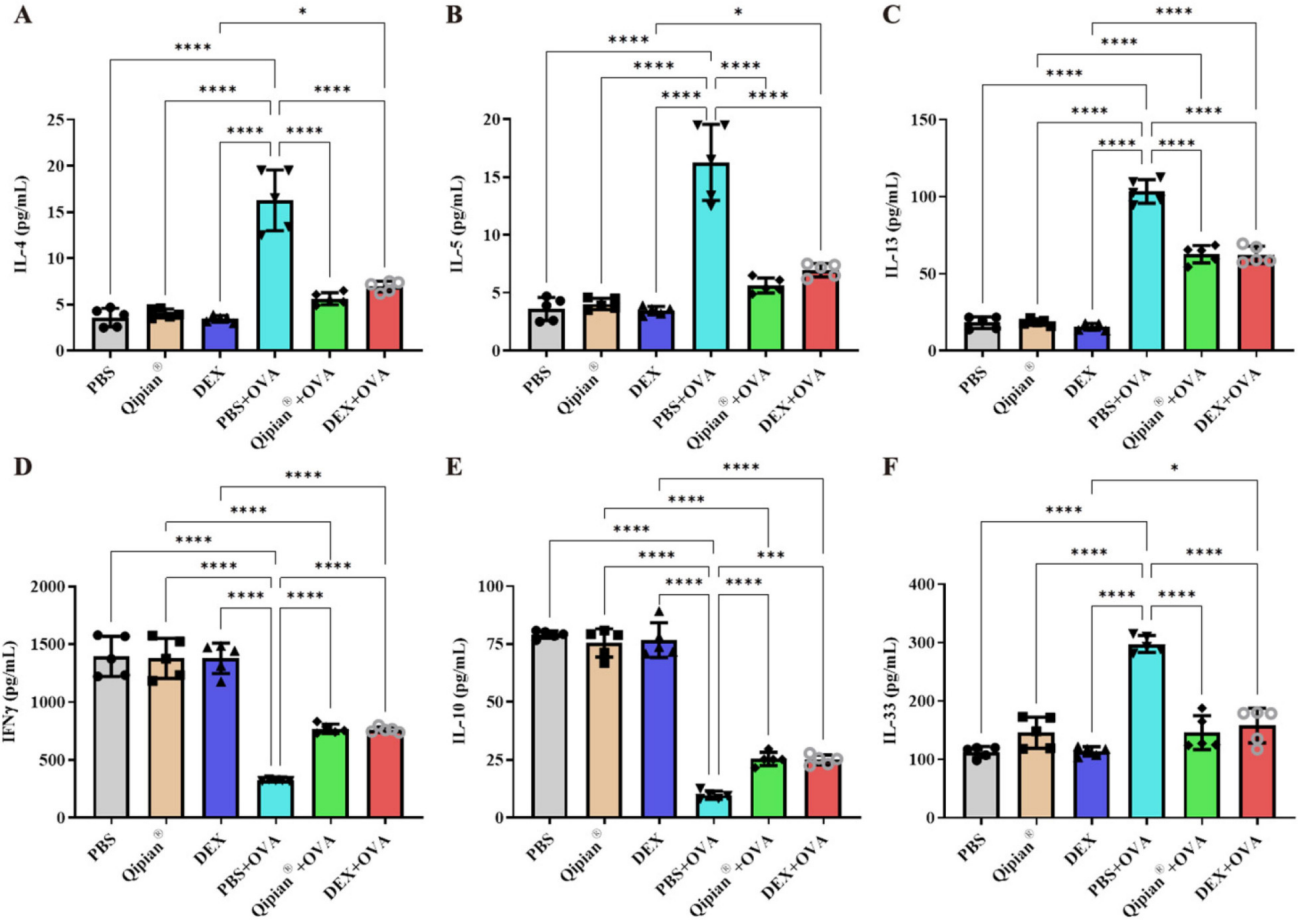
LTQA modulates cytokine levels in BALF. Concentrations of **(A)** IL-4, **(B)** IL-5, **(C)** IL-13, **(D)** IFN-γ, **(E)** IL-10, and **(F)** IL-33 were measured. Statistical significance is denoted as *p < 0.05, **p < 0.01, ***p < 0.001, and ****p < 0.0001.

CD4^+^ T cells, particularly Th2 cells, are essential effector cells that orchestrate the immune response in asthma (7). We therefore examined the impact of LTQA on the secretion of Th1/Th2 cytokines in BALF. The results demonstrated that LTQA effectively mitigated the OVA-induced elevation of Th2 cytokines, including interleukin-4 (IL-4), IL-5, and IL-13 (Figures 3A–C). Conversely, LTQA increased concentrations of Th1 cytokine IFN-γ and the regulatory cytokine IL-10 (Figures 3D,E). IL-5 plays a significant role in airway inflammation by regulating the recruitment, activation, and survival of eosinophils, which release various pro-inflammatory mediators (31). The suppressive effect of LTQA on IL-5 was consistent with the observed reduction in BALF eosinophils (Figure 1F). Furthermore, as the IL-33/ST2 pathway is known to drive type 2 immunity and allergic pathology (32, 33), LTQA also reduced the IL-33 release compared to the OVA model group (Figure 3F).

### 3.4 The LTQA promotes the expansion of CD4^+^ CD25^+^ Foxp3^+^ treg cells and the proliferation of CD103^+^ DCs

**Figure 4:**
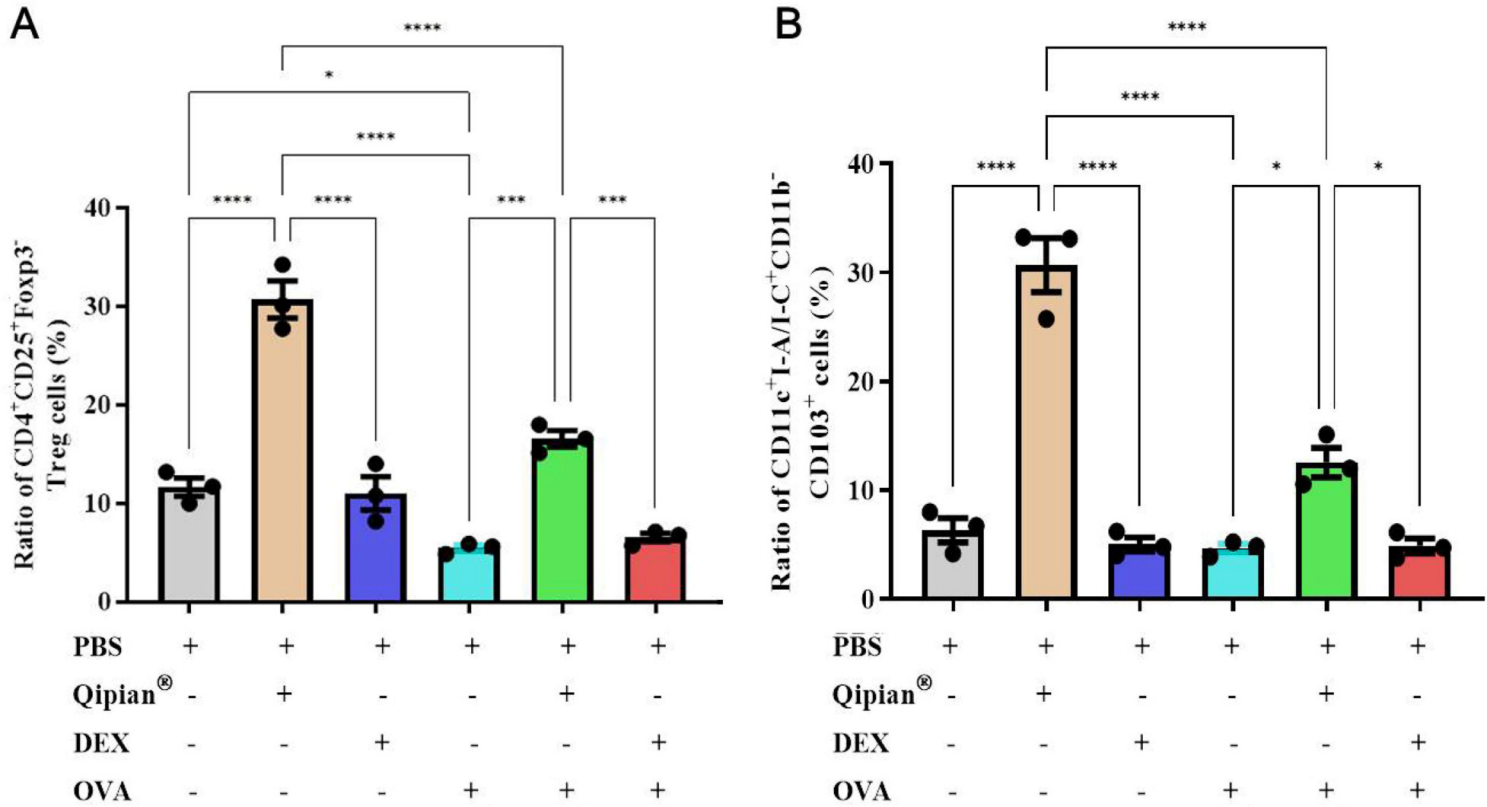
LTQA treatment is associated with increased frequencies of regulatory T cells and CD103^+^ dendritic cells. **(A)** Proportion of CD4^+^ CD25^+^ Foxp3^+^ regulatory T (Treg) cells in lung tissue. **(B)** Proportion of CD11c ^+^ I-A/I-E ^+^ CD11b^−^ CD103^+^ dendritic cells (DCs). Data are presented as mean ± SEM. Statistical significance is denoted as *p < 0.05, ***p < 0.001, and ****p < 0.0001.

Regulatory T (Treg) cells, characterized by the expression of CD4, CD25, and Foxp3, are known to be pivotal in the pathogenesis of asthma (34). To investigate the immunomodulatory potential of LTQA, we assessed its impact on CD4^+^ CD25^+^ Foxp3^+^ Treg cells within lung tissue (Figure 4A). In non-asthmatic mice, LTQA treatment was associated with a significant increase in Treg cells compared to the PBS control (Qipian® group: 30.82% ± 4.36% vs. PBS group: 10.75% ± 1.41%, *p* < 0.01). Conversely, OVA challenge markedly reduced the Treg cell proportion (OVA group: 4.34% ± 0.51% vs. PBS group: 10.75% ± 1.41%, *p* < 0.001). Notably, LTQA administration to asthmatic mice restored this deficit, resulting in a significantly higher Treg level than in the PBS-treated OVA model (OVA + Qipian® group: 15.05% ± 1.35% vs. OVA + PBS group: 4.34% ± 0.51%, *p* < 0.05). In contrast, DEX treatment did not significantly alter Treg cell proportions in either healthy or OVA-challenged mice. These findings suggest that the alleviation of airway inflammation by LTQA may be associated with its ability to bolster the Treg cell population.

Furthermore, we examined CD103^+^ DCs, which are critical antigen-presenting cells implicated in priming the differentiation of naïve T cells into Tregs in mice (35, 36). Our data indicate that LTQA treatment significantly increased the frequency of CD11c^+^ I-A/I-E^+^ CD11b^−^ CD103^+^ DCs compared to both PBS and OVA control groups (Figure 4B). Given that this DC subset has been shown to promote the generation of CD4^+^ Foxp3^+^ Treg cells (36), we infer that the concurrent expansion of CD103^+^ DCs and Treg cells observed in our study may be interrelated. This correlative evidence aligns with established literature on the role of CD103^+^ DCs in Treg development. Importantly, CD103^+^ DCs are also known to influence Th1 and Th17 responses in the airways following allergen exposure (37). Thus, the increase in this DC subset may also relate to the observed shift toward a Th1-dominant cytokine profile (Figure 3), contributing to the restoration of Th1/Th2 balance.

### 3.5 LTQA effectively inhibits asthmatic behaviors and lung tachykinin levels

**Figure 5:**
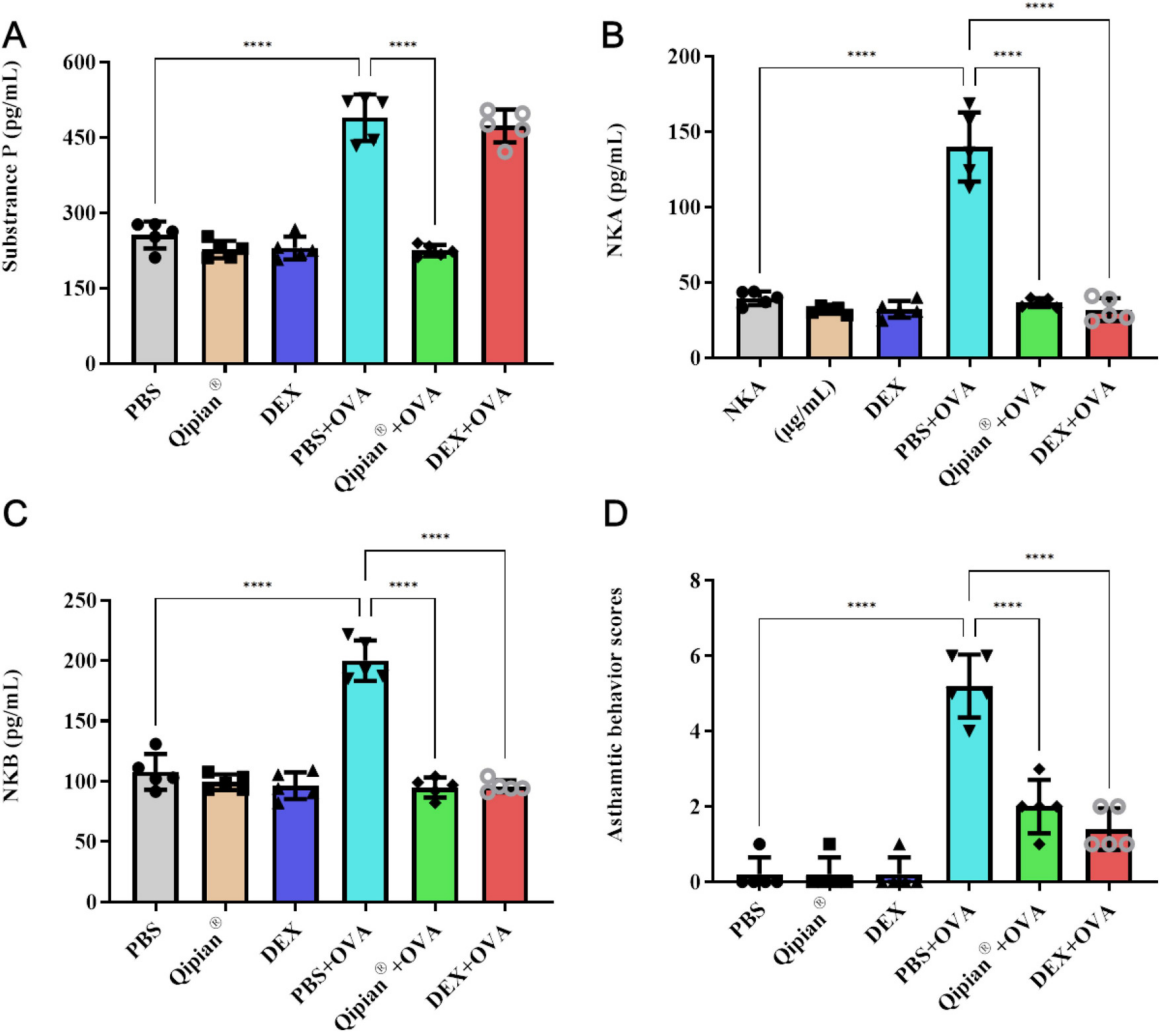
LTQA treatment is associated with reduced tachykinin levels and improved symptom scores in asthmatic mice. **(A–C)** Concentrations of substance *P* (SP), neurokinin A (NKA), and neurokinin B (NKB) in lung tissue. **(D)** Asthma-like behavioral scores. Mice were treated as described in Figure 4. Data are presented as mean ± SEM. Statistical significance vs. the OVA model group is denoted as *p < 0.05, **p < 0.01, ***p < 0.001, and ****p < 0.0001.

Eosinophils can raise airway sensory nerve (ASN) density in asthma (38). When activated by inflammatory mediators from immune cells, ASNs release tachykinins (SP, NKA, and NKB) (39). Our investigation revealed that OVA challenge significantly elevated pulmonary tachykinin levels, an effect that was attenuated by both LTQA and DEX (Figures 5A–C). LTQA pretreatment also inhibited the rise in SP. Concomitantly, both treatments reduced asthma symptom scores (Figure 5D). Collectively, these findings indicate that LTQA's beneficial effects are correlated with reduced tachykinin signaling. This modulation may be a direct effect or, more likely, a secondary consequence of the overall suppression of eosinophilic and Th2 inflammation.

### 3.6 LTQA impacts gut microbiome

**Figure 6:**
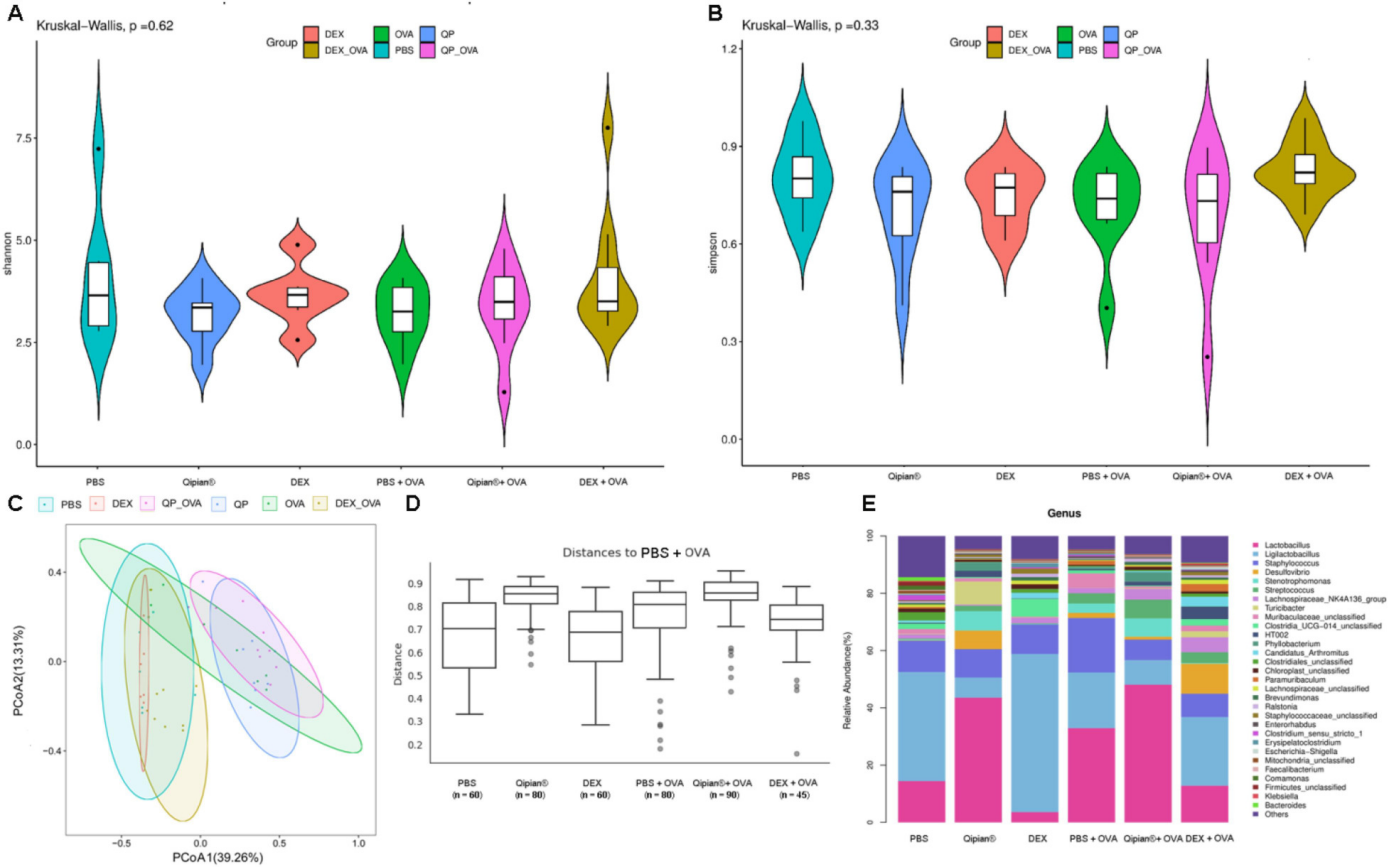
Microbial community analysis reveals LTQA-associated remodeling supporting the lung–gut axis. Microbial diversity and composition in fecal samples were analyzed via 16S rRNA gene sequencing. Shown are **(A,B)** alpha diversity indices, **(C)** a PCoA plot of beta diversity (Bray–Curtis dissimilarity), **(D)** ANOSIM results quantifying intergroup separation, and **(E)** the relative abundance of the 30 most prevalent genera. Error bars represent mean ± SEM. The coordinated shifts in microbial ecology following LTQA treatment correlate with improved asthma phenotypes, lending supportive evidence for gut–lung communication.

Mounting evidence suggests that the homeostasis and dysbiosis of intestinal flora are associated with protection and pathogenesis in asthmatic patients and mice (40, 41). Some BLs could also protect the host from asthma by promoting timely maturation of the gut microbiome (8). To investigate whether LTQA exhibits its antiasthma activity via modulation of the gut microbiome in the asthma model, bacterial 16S rDNA sequencing was conducted. At the genus level, there were no significant differences observed in the *α*-diversity of gut microbiota among mice in the six groups (Figures 6A,B) based on the Shannon and Simpson indices. PCoA and analysis of similarities (ANOSIM) of Bray–Curtis distances at the OTU level revealed significant differences in β diversity among the six groups (Figures 6C,D), with distinct bacterial flora distributions observed between the normal mice group and the OVA-induced asthmatic group (Figure 6E, *p* < 0.05). Among the top 30 genera of the gut microbiota, LTQA increased the abundance of *Lactobacillus* and *Stenotrophomonas* and reduced the abundance of *Ligilactobacillus* and *Desulfovibrio*, whereas DEX treatment increased the ratio of *Ligilactobacillus* and *Clostridia_UCG−014* and reduced the abundance of *Lactobacillus* compared to the model group (Figure 6E). Together, these analyses demonstrate an association between LTQA treatment and specific shifts in the gut microbial community, providing correlative support for its proposed systemic effects via the lung–gut axis.

**Figure 7:**
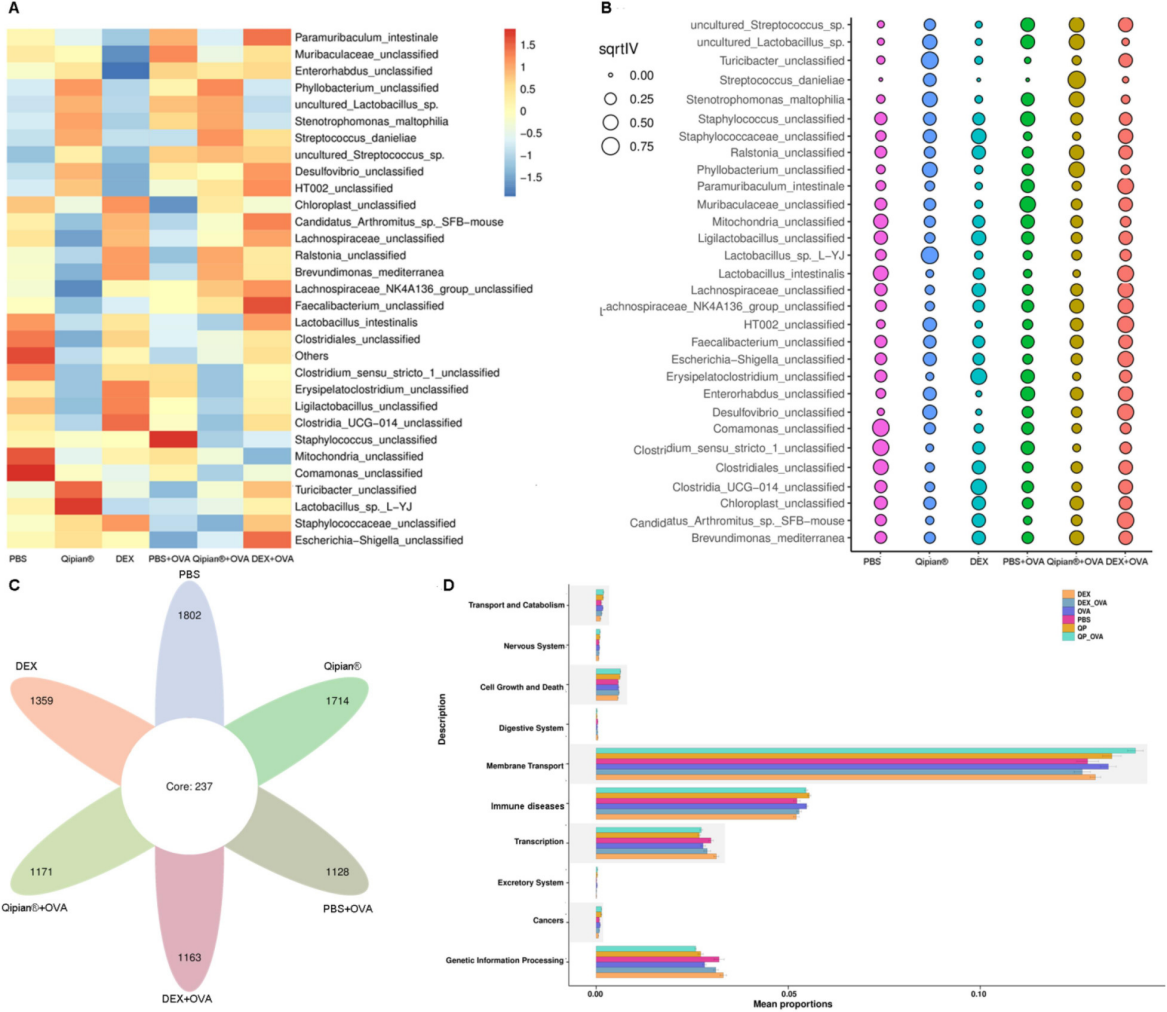
The analysis of biomarker features and functional prediction of gut microbiota via 16S rDNA sequencing: **(a)** assessing the relative abundance of gut microbiota species. **(B)** Generating a bubble chart using Random Forest and Gini index at the species level. **(C)** Conducting a Venn analysis to compare ASV profiles among six groups. **(D)** Predicting functions based on KEGG pathway analysis using Tax4Fun.

The composition of the gut microbiota was significantly altered by both asthma induction and therapeutic intervention. PCoA revealed clear separation between groups (Figures 7A,B). Differential abundance testing identified specific taxa associated with each condition. Ovalbumin challenge significantly increased the relative abundance of *Staphylococcus* and decreased *Comamonas* and certain *Clostridiales* compared to healthy control (*p* < 0.05). LTQA treatment significantly modulated the OVA-perturbed microbiota, characterized by an increase in *Lactobacillus* sp. L-YJ and *Streptococcus danieliae* and a decrease in several *Clostridia*-associated taxa. Dexamethasone induced a third unique profile, enriching for *Turicibacter* and *Lachnospiraceae*. Furthermore, microbial alpha diversity, measured by ASV richness, was significantly reduced in the asthmatic model (Figure 7C). To infer potential functional consequences, we used Tax4Fun to predict KEGG pathway abundances. The predicted metagenome of OVA-challenged mice was enriched for pathways involved in core metabolic processes (e.g., energy metabolism, transcription), whereas that of LTQA-treated mice was enriched for pathways involving environmental information processing (e.g., membrane transport) (Figure 7D). It is important to note that these are *predictions* based on 16S rRNA gene sequences. They suggest hypotheses for microbial metabolic shifts that require direct metagenomic or metabolomic validation.

## 4 Discussion

Our findings suggest that LTQA may help mitigate the development of asthma by counteracting the OVA-induced Th2-dominant immune response. This aligns with clinical studies showing that Qipian® can treat pediatric asthma by modulating the Th1/Th2 balance (17). In contrast to reports that certain BLs promote systemic Th1 responses in OVA-immunized mice (42), our study found that the LTQA did not alter the baseline Th1/Th2 cytokine balance in non-asthmatic mice. This suggests that its action may involve context-dependent immunomodulation rather than indiscriminate immune stimulation. The observed immune changes in healthy mice could reflect a state of baseline modulation or “immune training,” preparing the immune system to respond appropriately upon allergen encounter, independent of overt inflammation. However, it effectively alleviated the Th1/Th2 imbalance induced by OVA sensitization and challenge. Furthermore, LTQA demonstrated a regulatory impact on the humoral immune response in asthmatic mice, significantly reducing the concentrations of IgE and IgG in both serum and BALF. Given that Th2 cytokines (e.g., IL-4, IL-5) and IL-33 are known to drive isotype switching to IgE (32, 33), and that targeting Th2 cells reduces pathogenic IgG and IgE synthesis in asthma (43), these data indicate that LTQA's relief effect involves modulating key pathways in allergic sensitization. Importantly, DEX was used here as a standard-of-care therapeutic benchmark to suppress established inflammation. Its distinct mechanism—broad immunosuppression without the Treg/DC expansion seen with LTQA—underscores the fundamentally different immunological pathways engaged by the two interventions. This contrast highlights that LTQA's activity may extend beyond anti-inflammation to include positive immunomodulation and tolerance-promoting mechanisms.

The prevention of this immune imbalance by LTQA may be linked to its impact on CD4^+^ CD25^+^ Foxp3^+^ regulatory T (Treg) cells, which are critical modulators of both cellular and humoral immunity in asthma (44). Notably, children with asthma show reduced Treg cell proportions (45), and BL treatments have been shown to increase CD4^+^ CD25^+^ Foxp3^+^ lymphocytes in both asthmatic children (46) and the respiratory mucosa of asthmatic mice (36). In our study, LTQA likely increased Treg cells via the activation of CD103+ DCs. CD103^+^ DCs are a migratory population, promoting immune tolerance in allergic settings by inducing Treg differentiation (7). Our prior research showed that LTQA treatment markedly increased both Treg cells and CD103^+^ DCs in the lungs. This mechanistic profile appears to differ from that of other BLs such as Broncho-Vaxom (36), suggesting variations due to formulation, dosage, or specific mode of action. Qipian®'s ability to modulate the antigenic immune response may shift the course of asthma toward immune equilibrium, contrasting with the broad immunosuppressive effects of DEX therapy. Clinical studies support Qipian®'s efficacy in pediatric asthma (17, 47). However, the immunoregulatory role of BLs on DCs can vary significantly based on bacterial strains and dosages (17, 47), warranting further investigation to optimize therapeutic protocols. Finally, while other compounds such as MV-130 and OM-85 show promise through distinct immunomodulatory mechanisms (48, 49), and mixed lysates often exhibit synergistic efficacy (50), Qipian® demonstrates a particular strength in expanding CD103^+^ DC and Treg populations. Its capacity to directly stimulate Th1 responses, however, appears less potent compared to some other agents under investigation.

Qipian® in the OVA-induced model appears to extend beyond the established modulation of Th1/Th2 balance and Treg expansion, as evidenced by its association with altered levels of tachykinins (SP, NKA, and NKB) in BALF. Asthma symptoms such as cough and wheezing are linked to tachykinins, which interact with neurokinin receptors (e.g., NK-1) on vagal afferent fibers during airway inflammation (51). These neuropeptides, released from sensory nerve endings, can induce bronchoconstriction and cough via NK1, NK2, and NK3 receptors (51), while SP can also trigger inflammatory pathways in airway epithelial cells (52). NKA acts as a potent bronchoconstrictor with potential roles in lung development (53). In this study, LTQA treatment significantly attenuated the OVA-induced elevation of NKA, NKB, and SP levels, correlating with the alleviation of asthmatic symptoms. This suggests that tachykinin modulation is part of LTQA's broader therapeutic profile, though it remains to be determined whether this reflects a direct effect on neurogenic pathways or a secondary consequence of its primary immunomodulatory action.

Our study also revealed that LTQA treatment altered gut microbiome composition, a finding that may help explain its broader immunomodulatory effects. Notably, the LTQA group showed an increased abundance of *Lactobacillus* compared to the OVA-induced asthma group. As specific *Lactobacillus* strains have been shown to ameliorate airway inflammation and restore microbial homeostasis in both animal models and clinical settings (54, 55), this shift is of particular interest. The beneficial effects of LTQA, which extended beyond modulating Th2 responses to include the prevention of airway function decline, were associated with this increased resilience of the gut microbiome. These results align with the concept of a gut–lung axis, suggesting a bidirectional relationship between intestinal microbiota and pulmonary health. Collectively, our findings highlight the potential of targeting the gut microbiome as a therapeutic strategy for complex conditions like asthma.

## 5 Conclusions

In conclusion, our study demonstrates that LTQA has the potential to attenuate the development of OVA-induced asthma phenotypes. LTQA appears to help restore the Th1/Th2 balance by recruiting Tregs to the airways and inhibiting neurogenic inflammation. Notably, its therapeutic effect was associated with remodeling of the gut microbiota, including an increased proportion of probiotic bacteria, supporting the involvement of the lung–gut axis. Further investigations are warranted to fully elucidate the pathways mediating the antiasthmatic activities of Qipian® and to optimize dosing regimen. Overall, our results provide valuable insights into the potential of LTQA as a strategy for alleviating asthma in a clinical context.

## Data Availability

The original contributions presented in the study are included in the article/Supplementary Material, further inquiries can be directed to the corresponding authors.
